# Understanding the Effect of Electron Beam Melting Scanning Strategies on the Aluminum Content and Materials State of Single Ti-6Al-4V Feedstock

**DOI:** 10.3390/ma16196366

**Published:** 2023-09-23

**Authors:** Katie O’Donnell, Maria J. Quintana, Peter C. Collins

**Affiliations:** 1 Department of Materials Science and Engineering, Iowa State University, Ames, IA 50011, USA; katieo1@iastate.edu (K.O.); mariaqh@iastate.edu (M.J.Q.); 2 Ames Laboratory, Ames, IA 50011, USA; 3 Department of Materials Science and Engineering, Carnegie Mellon University, Pittsburgh, PA 15213, USA

**Keywords:** additive manufacturing, preferential vaporization, composition, Ti-6Al-4V, feedstock materials

## Abstract

Research on the additive manufacturing of metals often neglects any characterization of the composition of final parts, erroneously assuming a compositional homogeneity that matches the feedstock material. Here, the composition of electron-beam-melted Ti-6Al-4V produced through three distinct scanning strategies (linear raster and two point melting strategies, random fill and Dehoff fill) is characterized both locally and globally through energy-dispersive spectroscopy and quantitative chemical analysis. As a result of the different scanning strategies used, differing levels of preferential vaporization occur across the various parts, leading to distinct final compositions, with extremes of ~5.8 wt.% Al and ~4.8 wt.% Al. In addition, energy-dispersive spectroscopy composition maps reveal specific features in both the XY and XZ planes (with Z being the build direction) as a result of local inhomogeneous preferential vaporization. The subsequent change in composition significantly modifies the materials’ state of parts, wherein parts and local regions with higher aluminum contents lead to higher hardness levels (with a ~50 HV difference) and elastic property values and vice versa. While varying scan strategies and scan parameters are known to modify the microstructure and properties of a part, the effect on composition cannot, and should not, be neglected.

## 1. Introduction

Additive manufacturing (AM) has been a heavily researched processing technique over the last few decades due to its many advantages over traditional processing techniques, including reduced waste and the ability to produce complex geometries [1,2,3,4,5,6]. Literature studies on AM have been primarily focused on obtaining the microstructure/texture and mechanical properties required for structural applications [7,8,9,10,11], neglecting other aspects of AM parts, such as composition. There is often an assumption that the final part will have the same composition as the feedstock material used in the process, with literature studies focusing on optimizing the mixing of powder and parameters to obtain the adequate melting of all powder components, including when delving into gradient materials [12,13,14]. However, there are thermodynamic phenomena applicable to AM that influence the final composition and that cannot, and should not, be ignored when using single feedstock powder.

Preferential vaporization is a well-studied phenomenon wherein, at high temperatures, elements with higher vapor pressures vaporize first, and at a faster rate than the other constituents of the material [15,16,17]. Examples of elements and alloy systems that are known to preferentially vaporize, both in conventional and additive manufacturing processes, include zinc and gallium, aluminum in titanium alloys, and chromium in stainless steels and nickel-based alloys [15,17,18]. If parameters for a process are already established, manufacturers can compensate for the known evaporation properties of high-vapor-pressure elements by increasing the quantity of these elements in the feedstock material, ensuring the final composition, despite element loss, is as desired [19,20]. For processes that are not established, an in-depth understanding of, and an ability to control, the temperatures reached during processing would also be beneficial in limiting preferential vaporization, given the dependence on temperature, as per the Langmuir equation [6,15,16]. Another process parameter that can affect vaporization, in AM in particular, is the pressure of the chamber, wherein higher chamber pressures limit evaporation [16,17,21]. Post-processing homogenization heat treatments can also be employed to mitigate any local variations in composition within a part, though the lost composition cannot be recovered. However, preferential vaporization is sometimes desired, as it can be used to remove impurity elements, such as oxygen in titanium alloys and inclusions [22].

Aluminum is a strong α stabilizer used in titanium alloys, influencing the microstructure (e.g., phase fractions and α lath sizes), thermophysical properties (e.g., β transus temperature, density, and viscosity), and mechanical properties (e.g., elastic modulus, yield strength, and hardness) [23,24,25,26]; therefore, understanding the preferential vaporization of Al is critical in defining the final materials state of AM parts. The rate of Al vaporization is heavily influenced by melt pool morphology and thermal gradients, which are themselves controlled by processing parameters (including scanning strategy), suggesting that a deeper understanding of these parameters could be conducive to the local or global compositional control of AM parts.

Beyond preferential vaporization, processing parameters (such as scan strategies) have an effect on other aspects of the materials state of final parts [27,28,29,30,31,32,33,34,35,36]. Changes in microstructure, texture, and mechanical properties, as well as percent porosity, for example, follow changes in processing parameters, including scanning strategy. Point melting scan strategies have shown promise in reducing the porosity of AM parts [28,37]. In Ti-6Al-4V samples, point melting strategies, as opposed to the more common linear raster scan strategies, typically result in slower cooling rates, leading to coarser as-built microstructures with higher fractions of colony microstructures [28,29,31,32,33,36]. However, to the authors’ knowledge, the effect of point melting on composition is yet to be explored.

This study analyzes compositional variations in electron-beam-melted (EBM) Ti-6Al-4V produced using three distinct scanning strategies from a single feedstock, including a single linear raster scan strategy and two variations on point melting. The significance of these variations is then explored by examining the elastic and plastic properties of the builds.

## 2. Materials and Methods

Electron-beam-melted Ti-6Al-4V samples were created in two separate builds using an ARCAM EBM Q10plus system with a stainless steel build plate. The parts were built at Oak Ridge National Laboratory’s Manufacturing Demonstration Facility. Both builds used the same general parameters, with a layer thickness of 50 µm, a preheat temperature of 470 °C, and a chamber pressure of 4.5 × 10^−2^ mBar. All samples analyzed here had a geometry of 15(X) × 15(Y) × 25(Z) mm, across both builds. Three different scan strategies were used: raster (L), random (R), and Dehoff (D). Random and Dehoff are both point melting scan strategies, while the raster is a linear melting scan strategy. Batch one of the two builds was created using TEKNA-plasma-atomized Ti-6Al-4V powder (with diameters between 45 and 105 µm) and samples were generated with all three scan strategies, designated as L5 (raster), R5 (random), and D5 (Dehoff). Batch two was built with AP&C-plasma-atomized Ti-6Al-4V powder (with the same diameter range as the previous batch), and only contained samples built with the raster and random scan strategies.

**Figure 1:**
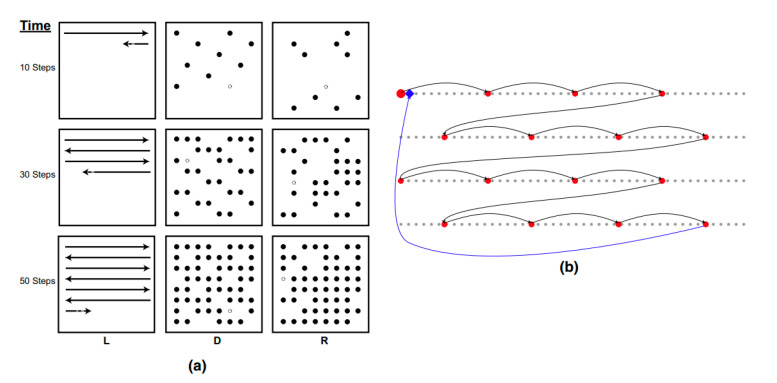
Schematic of the different scanning strategies at different time steps. L is a raster-line scan, D is a Dehoff point melting strategy, and R is a random point melting strategy (**a**). The open circle and smaller line represent the position that will be active in one additional time step. Representation of the first steps of the Dehoff strategy (**b**), with the diamond showing where the next melting point would be. The figure and caption are reprinted with permission from Ref. [36].

Parameters for each scan strategy were kept the same between the batches. More information on these parameters can be found in [28,34,36,37], but can be summarized as a beam current of 28 mA for L and a beam current of 11 mA for R and D; L used a travel speed of 4550 mm/s, while D and R had a residence time of 0.3 ms/spot. For the random samples, the point melting fill was randomly generated using a MATLAB random function to ensure that every location was melted once, while for the Dehoff scan strategy, an ordered fill melted each eleventh point in a row, skipped down five rows, and resumed melting every eleventh point, until every location was melted once (Figure 1). The parts were removed from the build plates with electrical discharge machining and prepared with typical metallographic procedures for titanium samples. 

Energy-dispersive spectroscopy (EDS) was used to obtain semi-quantitative local compositional information and composition maps from the batch one samples. EDS hardware and AZtec software (AZtec version 3.3 SP1) were obtained from Oxford Instruments, attached to an FEI Teneo LoVac field emission scanning electron microscope (SEM). Chemical analysis was performed at Luvak Laboratories on unpolished cut samples from batch two. Carbon was quantified using combustion infrared detection, as per ASTM E 1941-16. Oxygen and nitrogen were quantified using combustion infrared detection, as per ASTM E 1409-13. All other elements were quantified using direct current plasma emission spectroscopy, as per ASTM E 2371-13. The values for all elements were rounded, as per ASTM E29.

Density measurements were performed using the Archimedes method [38] and ultrasonic testing was performed with a 5 MHz transducer to collect both longitudinal and shear waveforms on the samples from batch two. Hardness testing was performed on the batch one samples using a zwickiLine Z2.5+ Hardness Tester. Vickers microhardness measurements were performed with a load of 200 g and a dwell time of 12 s. Measurements of the diagonals of the resulting indents were performed automatically using the testXpert software (Version 12.6) included with the zwickiLine machine; select indentations that fell outside of the range of plus or minus one standard deviation from the average were re-measured manually using the same software and optics. 

## 3. Results and Discussion

**Figure 2:**
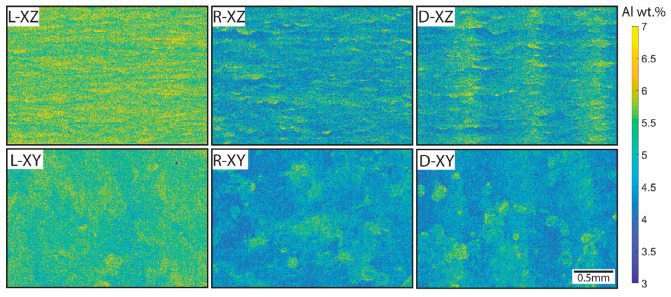
Energy-dispersive spectroscopy (EDS) maps depicting the aluminum content across orientations and scan strategies of the Ti-6Al-4V AM samples restricted from 3 to 7 wt.% Al (L: raster; R: random; D: Dehoff). As vanadium content remained consistent throughout the samples, EDS maps depicting this element are not shown.

Initial compositional analysis was performed via the EDS mapping of both the XZ and XY planes of the batch one samples (Figure 2). From the EDS maps, a difference in composition between the samples was evident, with the raster scan strategy resulting in the highest aluminum content (more yellow/lighter, closer to the 6–7 wt.% range) and the random scan strategy resulting in the lowest aluminum content (more blue/darker, closer to the 4–6 wt.% range) in the final parts. Not only did the composition vary globally between the parts, but the Al content varied locally based on the scan strategy as well. Within the raster scan strategy maps, horizontal bands of ‘high’ and ‘low’ aluminum could be observed in the XZ plane, leading to a relatively uniform composition in the XY plane map. Distinct melt pools in both planes could be distinguished more easily in the point melting scan strategies (R5 and D5). The randomness versus the ordered nature of the two point melting scan strategies could also be seen when comparing the R5 and D5 maps, wherein distinct vertical ‘stripes’ of high and low Al spanned both the XY and XZplanes in the D5 sample.

A semi-quantitative assessment of aluminum content from a compilation of EDS analyses is presented in Table 1. Column one of the data presented in in Table 1 shows the averaged aluminum content of the XY and XZ maps shown in Figure 2. The second column averages the aluminum content from smaller-scale EDS maps near the defects, as published in [39,40,41]. Data in the third column are taken from EDS area analyses conducted on the centerline of each of the samples. Finally, the fourth column presents data from smaller EDS area analyses near the hardness indents that will be discussed later in this study. The differences in aluminum content between the scan strategies can be elucidated further from these results compared to what can be qualitatively seen in Figure 2. The raster scan strategy sample retains the most Al during the EBM process, while the random scan strategy sample has nearly 0.65 wt.% less Al. The Dehoff scan strategy, meant to be a middle ground between the ordered linear scans of the raster sample and the sporadic point melt pools of the random sample, contains slightly more Al than the latter.

The quantitative chemical analysis results are presented in Table 2. While the magnitude of the aluminum content varied slightly from that observed in the semi-quantitative EDS scans (as expected due to the differences between techniques), the variation between the samples did not. The raster scan strategy sample had a composition of 5.88 wt.% Al, while the random scan strategy sample had a composition of 4.87 wt.% Al, meaning ~1 wt.% Al less. Vanadium, in contrast, was similar between the two samples, confirming the expectation that this element does not experience preferential vaporization, or does at a lesser degree than Al [15]. Thus, the random scan strategy sample falls outside the composition bounds of aluminum content for Ti-6Al-4V, and the Dehoff scan strategy sample can be expected to follow, based on the EDS results. Despite being printed with the same feedstock material as part of the same build, one sample maintained the starting composition (raster) and another sample did not (random).

Given the difference in chemical composition between the three scan strategies, a difference in properties and materials state was expected, and tests were conducted to examine the significance of the differences. Firstly, density measurements were performed on the batch two samples to obtain the elastic behavior from ultrasonic testing. Both longitudinal and shear waveforms were collected from the raster and random samples, from which, along with the density and thickness measurements of the samples tested, the elastic modulus, Poisson’s ratio, shear modulus, and bulk modulus can be calculated using Equations (1)–(4). The results of this are displayed in Table 3.

(1) $$E=\rho V_{S}^{2}\left(3V_{L}^{2}-4V_{S}^{2}\right)/\left(V_{L}^{2}-V_{S}^{2}\right)$$

(2) $$\nu =\left[{1-2\left(V_{S}/V_{L}\right)}^{2}\right]/\left[{2-2\left(V_{S}/V_{L}\right)}^{2}\right]$$

(3) $$G=V_{S}^{2}\rho$$

(4) $$K=\rho \left[V_{L}^{2}-\left(4/3\right)V_{S}^{2}\right]$$

where E is the elastic modulus, ν is Poisson’s ratio, G is the shear modulus, K is the bulk modulus, ρ is the density of the sample, $V_{s}$ is the shear wave velocity, and $V_{L}$ is the longitudinal wave velocity. Longitudinal and shear velocities are calculated as being double the thickness of the sample divided by the time of flight between successive echoes from the ultrasonic waveforms.

The presence of a higher aluminum content in the raster scan strategy samples resulted in higher values for all elastic properties measured or calculated. Given the relationship of Al to elastic properties [24,42,43], the results were expected, except for the difference in density between the samples. A higher overall Al content was expected to produce a lower overall density, the opposite of what was observed here. An explanation for the density differences between the scan strategies goes beyond the scope of this paper; however, differences in the phase fraction (as the different crystal structures and lattice parameters of α and β result in different densities), also affected by composition, likely play a role. 

To examine the mechanical property differences, Vickers microhardness traverses were conducted on both the XY and XZ planes of each of the three scan strategies from the batch one samples, in addition to a single wrought Ti-6Al-4V sample for comparison purposes. For the XY planes and the wrought sample, traverses consisted of 64 indents in an eight-by-eight indent grid. For the XZ planes, the traverses covered from the bottom to the top of the build height in three columns of 45 indents each, for a total of 135 indents per traverse. Statistical results from each traverse and scan strategy are presented in Table 4. As expected, the raster sample (with the highest aluminum content) had the highest hardness, while the random sample (with the lowest aluminum content) had the lowest hardness. The Dehoff sample again fell between the two, and had slightly higher standard deviations, as portions of the sample varied periodically in aluminum content. In comparison to the wrought sample, the spread of the data remained roughly the same from sample to sample, demonstrating that the variation between the AM samples was significant.

**Figure 3:**
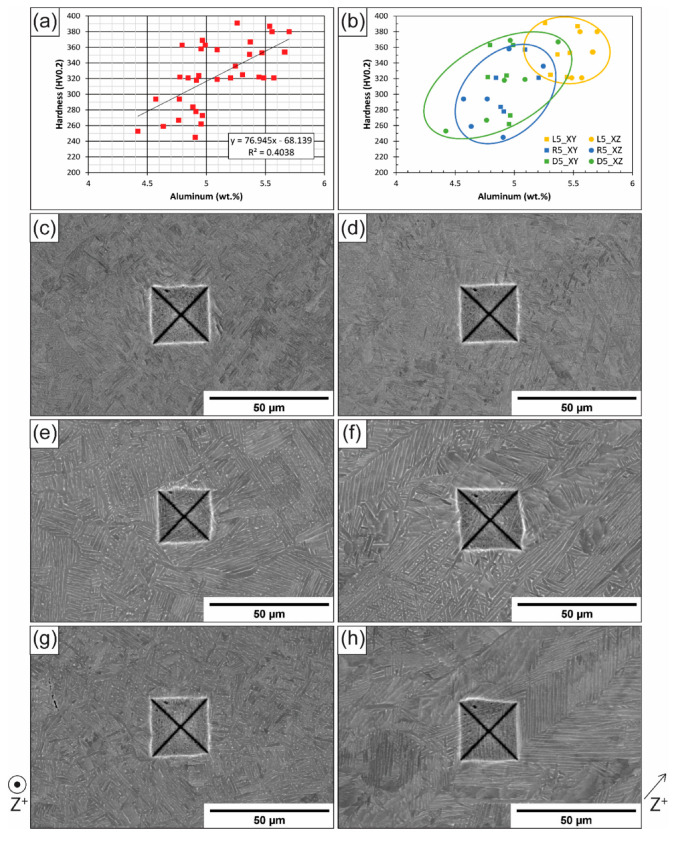
Hardness–composition correlation results showing (**a**) all hardness and aluminum results plotted with a linear trendline and (**b**) all results plotted again, this time with distinctions between each sample and region from which each data point was collected, and images of representative indents from (**c**) the XY and (**d**) XZ plane of L5, (**e**) the XY and (**f**) XZ plane of R5, and (**g**) the XY and (**h**) XZ plane of D5. The build direction for images (**c**,**e**,**g**) is indicated on the lower left of the figure; the build direction for images (**d**,**f**,**h**) is indicated on the lower right of the figure.

To further understand the correlation between hardness and Al content, small-scale EDS analyses were conducted on the sides of indents with the highest, lowest, and average hardness values (six in total) for each plane of the three AM samples. As a result, 36 local Al measurements were taken and compared with the relevant hardness values. The results are plotted in Figure 3a,b, with Figure 3a displaying the linear trendline and R^2^ values for the entire dataset (all samples) and Figure 3b distinguishing between the scan strategies and planes tested. The Pearson correlation coefficient R value of the overall correlation was 0.6355 (with an R^2^ of 0.404, as shown in Figure 3a), and the *p*-value was 0.000041. Knowing that an R value of 1 is a perfect positive correlation and −1 is a perfect negative correlation, the R value showed a moderate positive correlation between the two variables (hardness and aluminum content). Considering the *p*-value, anything less than 0.05 was considered statistically significant. Given the *p*-value for this correlation, the data strongly suggest that the relationship between hardness and aluminum content is not random.

The relationship between aluminum content and mechanical properties, including hardness, is primarily driven by solid solution strengthening, wherein both theory and models have suggested a power law relationship, with an exponent on the Al content variable ranging from ½ to ⅔ [24,25]. The data presented here are consistent with those of solid solution strengthening models, but to better examine the relationship between hardness and Al content, exponents ranging from 0.5 to 1 were examined for this dataset. There were minimal changes in the resulting R^2^ value; for an exponent of 1, as depicted in the trendline in Figure 3, recall for R^2^ was 0.404, while for an exponent of 0.5, the subsequent R^2^ value was 0.406. As such, the data here are insufficient in precisely determining the exponent in a power law relationship, though such a task would be difficult to perform from the hardness testing results alone.

In addition to the correlation between hardness and aluminum content, differences in microstructure between the scan strategies can also be clearly seen in Figure 3c–h, which display selected hardness indents. Qualitatively speaking, the random scan strategy sample had the largest lath sizes and the highest fraction colony, while the raster scan strategy sample had the smallest laths and the lowest colony fraction. While a qualitative assessment of the microstructure was not conducted as part of this study, previous analyses have been published on these samples [36], as well as on other samples in the same build [29,31,32,44,45,46,47], supporting qualitative conclusions. The difference in microstructure was primarily a result of cooling rate differences, with the raster sample cooling the fastest, enabling the formation of a basketweave structure, and the random sample cooling the slowest [36], forming more colony. A deconvolution of the exact influence of the cooling rate and aluminum content differences on microstructure, as well as between the effect of microstructure and aluminum content differences on hardness, goes beyond the scope of this study.

## 4. Conclusions

The results presented in this study show that:

- Using Ti-6Al-4V powder does not always translate to Ti-6Al-4V parts. While the raster scan strategy results in a Ti-6Al-4V sample, random and Dehoff strategies result in a composition that can be approximated to Ti-5Al-4V. As such, these samples would not be able to be certified as grades 5 (aluminum content between 5.50 and 6.75 wt.%) or 23 (aluminum content between 5.50 and 6.50 wt.%).
- The aluminum difference between samples results in raster samples (with higher Al contents) with higher values for density; longitudinal and shear velocities; elastic, bulk, and shear moduli; Poisson’s ratio; and hardness.
- In addition, EDS maps are clearly distinct between scanning strategies, allowing for the identification of each sample through compositional patterns alone, where point melting scan strategies show individual melt pools, while the raster scan strategy shows horizontal banding in the XZ plane and the Dehoff sample shows vertical ‘stripes’ of varying Al content.

## Figures and Tables

**Table 1:** Comparison of the aluminum weight percent values from a variety of EDS analyses conducted on different areas of the batch one Ti-6Al-4V samples.

Sample	Large-Scale EDS Maps (Figure 1)	Small-Scale EDS Maps	Centerline Area Analyses	Hardness Area Analyses	Average
Raster (L5)	5.48 ± 0.25	5.40 ± 0.20	5.54 ± 0.04	5.50 ± 0.16	5.47 ± 0.17
Random (R5)	4.63 ± 0.11	4.66 ± 0.20	4.58 ± 0.01	4.89 ± 0.25	4.83 ± 0.26
Dehoff (D5)	4.72 ± 0.20	4.87 ± 0.23	4.80 ± 0.08	4.91 ± 0.26	4.89 ± 0.25

**Table 2:** Chemical analysis of raster and random samples.

	Raster	Random
Carbon	0.019	0.023
Nitrogen	0.012	0.013
Oxygen	0.139	0.161
Aluminum	5.88	4.87
Silicon	0.014	0.014
Titanium	Balance	Balance
Vanadium	4.18	4.27
Chromium	0.012	0.010
Iron	0.21	0.21
Nickel	0.0098	0.0094
Copper	0.0020	0.0020

**Table 3:** Elastic properties of the raster and random samples obtained using ultrasonic testing.

Sample	Density (kg/m^3^)	Longitudinal Velocity (m/s)	Shear Velocity (m/s)	Elastic Modulus (GPa)	Poisson’s Ratio	Shear Modulus (GPa)	Bulk Modulus (GPa)
Raster	4420	6220	3170	117.6	0.325	44.4	111.9
Random	4380	6120	3150	115.0	0.320	43.5	106.4

**Table 4:** Vickers microhardness results of the three samples (raster—L, random—R, and Dehoff—D) in two planes (XY and XZ), as well as a wrought Ti-64 sample.

Sample	Region	Vickers Hardness [HV0.2]		
		Average	Range	95% Confidence Interval
L5	XY Plane	353 ± 17.7	322–391	4.3
	XZ Plane	354 ± 14.9	321–380	2.5
	Overall	353 ± 15.8	321–391	-
R5	XY Plane	321 ± 17.7	278–378	4.4
	XZ Plane	294 ± 19.5	245–358	3.3
	Overall	303 ± 22.7	245–378	-
D5	XY Plane	322 ± 20.0	262–363	5.0
	XZ Plane	319 ± 23.3	253–369	3.9
	Overall	320 ± 22.3	253–369	-
Wrought Ti-6Al-4V	N/A	355 ± 17.1	320–389	4.2

## Data Availability

The data presented in this study are available upon request from the corresponding author. The data are not publicly available due to the ongoing nature of the project.
